# Cross-Sectional Survey of High-Risk Pregnant Women's Opinions on COVID-19 Vaccination

**DOI:** 10.1089/whr.2022.0006

**Published:** 2022-06-29

**Authors:** Marcia DesJardin, Edward Raff, Nicholas Baranco, Dimitrios Mastrogiannis

**Affiliations:** ^1^ Department of Obstetrics and Gynecology, SUNY Upstate, Syracuse, New York, USA.; ^2^ Booz Allen Hamilton, Baltimore, Maryland, USA.; ^3^ Department of Graduate Data Science, University of Maryland Baltimore County, Baltimore, Maryland, USA.

**Keywords:** COVID, pregnancy, vaccination

## Abstract

**Background:**

Pregnant women are at increased risk of severe disease with coronavirus disease 2019 (COVID-19). Despite strong recommendations from American College of Obstetricians and Gynecologists and Society for Maternal Fetal Medicine for vaccination, COVID-19 vaccination hesitancy persists. With this study, we aim to evaluate opinions about the COVID-19 vaccine in a cohort of high-risk pregnant patients.

**Materials and Methods:**

Institutional review board approval was obtained. Patients attending a regional Maternal–Fetal Medicine clinic in central New York were surveyed about the COVID-19 vaccine using a standardized questionnaire. Demographic, obstetrical, and medical information was abstracted using medical records. The vaccinated and unvaccinated groups were evaluated using chi-square tests and a Bayesian model.

**Results:**

Among the 157 participants, 38.2% are vaccinated. There were no significant differences in race/ethnicity, living situation, marital status, employment status, insurance type, pregravid body mass index, history of recreational drug use, number of living children, or gestational age at the time of survey. Patients with less formal education are less likely to be vaccinated. There was no difference between influenza and tetanus diphtheria pertussis vaccination rates with COVID-19 vaccination rates. Unvaccinated patients cite lack of data in pregnancy (66%) as their primary concern. Most patients prefer to learn about vaccines *via* conversation with their doctor (46.7% for vaccinated and 59.8% for unvaccinated).

**Conclusions:**

The vaccination rate is low in our population. A provider-initiated conversation about COVID-19 vaccination included with routine prenatal care could increase the vaccination rate.

## Introduction

Pregnant women are at increased risk of severe disease with infectious viruses.^1^ With coronavirus disease 2019 (COVID-19) infection, pregnant women are more likely to be admitted to an intensive care unit, require invasive ventilation, and die^2,3^ compared with nonpregnant subjects. Despite these statistics, there is widespread distrust with the vaccine and lack of vaccination in pregnant women. By May 2021, 46% of reproductive-aged women received one dose of the COVID-19 vaccine, whereas only 16% of pregnant women had done the same.^4,5^ The vaccination rate of pregnant women rose to only 33% by October 1, 2021 despite multiple efforts to improve access and information about the vaccination.^6–14^

Pregnant women were excluded from initial COVID-19 vaccination clinical trials.^1,12^ As a result, initial recommendations for the vaccines were mostly empiric for pregnant women, primarily based on observational real data and resulted in a great amount of confusion and concern (with the additive effect of politization).

The Centers for Disease Control (CDC) has an ongoing registry (V-Safe) to collect data regarding vaccination outcomes in pregnancy.^13^ Since the onset of V-safe, studies that compiled the results of the registry demonstrated safety in pregnancy.^14^ The American College of Obstetricians and Gynecologists (ACOG), Society for Maternal Fetal Medicine (SMFM), and CDC all published statements strongly in support of vaccination against COVID-19 in all women, including pregnant and lactating patients.^15–30^ The mistrust created by the early trial designs and exacerbated by social media leaves pregnant women and fetuses vulnerable during this pandemic.

COVID-19 infection has been shown to cause more severe disease in patients with existing comorbidities, such as obesity, hypertension, diabetes mellitus, and asthma.^31,32^ With the additional baseline risk of severe disease in pregnancy, there is an increasing risk of severe disease if infected with COVID-19 in pregnant patients. With this study, we aim to evaluate opinions about the COVID-19 vaccine in a sample of high-risk pregnant patients in central New York.

## Materials and Methods

**FIG. 1.**
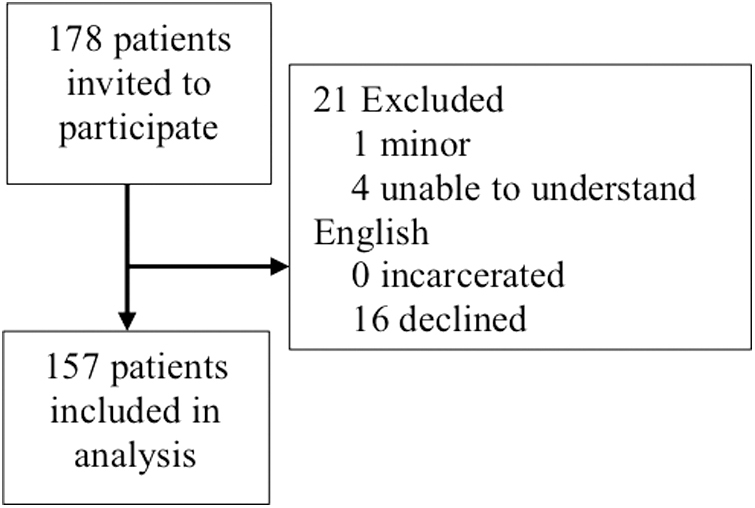
Prisma diagram.

Institutional review board approval was obtained (IRB No. 1722484-1, approved June 6, 2021). All patients who presented for prenatal care at a central New York regional Maternal–Fetal Medicine clinic September to October 2021 while a research staff member was present were invited to participate. Participants were excluded if they met exclusion criteria: age <18 years, unable to understand English, or incarcerated (Fig. 1). Convenience sampling was used due to ease of logistics with limited resources.

The research staff member administered the survey to all available participants for four sequential weeks during all clinic hours except Fridays before noon. After informed consent was obtained, patients answered a standardized survey about the COVID-19 vaccine and demographic information. The survey was modeled after information gathered by Kaiser Family Foundation for national general population trends about COVID-19 vaccination hesitancy.^33^ Participants were given a tablet to self-administer the written survey while a research staff member was available for clarification questions.

The survey was available only in English. Further demographic, obstetrical, and medical information was abstracted using medical records. Study data were collected and managed using REDCap (Research Electronic Data Capture) electronic data capture tools hosted by SUNY Upstate Medical University.^34,35^ The COVID-19 vaccination status was used to identify the primary outcome. The vaccinated and unvaccinated groups were evaluated with a hierarchical Bayesian model. An effect size was generated *via* the Bayesian model; a credible interval that did not include zero was considered statistically significant.

## Results

**FIG. 2.**
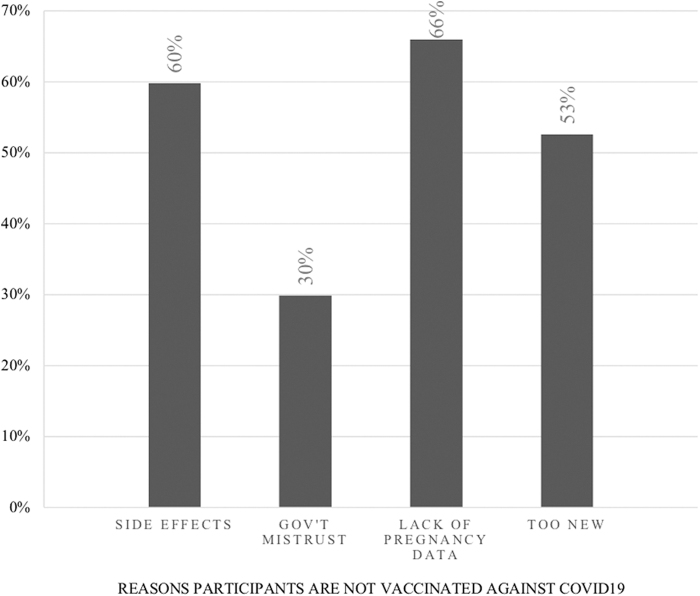
Reasons for lack of vaccination.

A total of 178 pregnant women were invited to participate in the study. Twenty-one participants were excluded; 16 declined to participate, 4 were unable to understand English, and 1 was <18 years old (Fig. 1). One hundred fifty-seven pregnant women were included in the analysis, for an 88% response rate (Table 1). All participants completed the survey provided. 38.2% reported that they received the COVID-19 vaccine. The most common reason for vaccine hesitancy was the lack of data about the vaccination in pregnancy (Fig. 2).

**Table 1.** Categorical Abstracted Information, by Coronavirus Disease 2019 Vaccinated Versus Not Vaccinated Status

Characteristics	Total (%)	Vaccinated, *n* (%)	Not vaccinated, *n* (%)	Bayesian model: effect size (95% CI)^a^
Total	157 (100%)	60 (38.2)	97 (61.7)	n/a
Race and ethnicity				
Asian	8	1 (12.5)	7 (87.5)	−1.04 (−3.05 to 1.07)
Non-Hispanic White	116	49 (42.4)	67 (57.8)	0.85 (−1.03 to 2.67)
Hispanic	8	5 (62.5)	3 (37.5)	1.02 (−1.03 to 3.09)
Non-Hispanic Black	17	2 (11.8)	15 (88.2)	−0.33 (−2.38 to 1.69)
American Indian/Alaskan Native	2	0 (0)	2 (100)	−0.54 (−2.83 to 1.68)
Other	5	2 (40)	3 (60)	0.24 (−1.68 to 2.24)
Living situation				
Own home	66	32 (48.5)	34 (51.5)	0.12 (−1.81 to 1.94)
Rent home	73	21 (28.8)	52 (71.2)	−0.27 (−2.09 to 1.55)
Family household	18	7 (38.9)	11 (61.1)	−0.12 (−1.99 to 1.81)
Employment status				
Full time	76	31 (40.8)	45 (59.2)	−0.30 (−2.07 to 1.56)
Part time	18	11 (61.1)	7 (38.9)	0.73 (−1.21 to 2.65)
Other	11	3 (27.2)	8 (72.7)	−0.98 (−2.92 to 0.94)
Unemployed	44	13 (29.5)	31 (70.5)	−0.28 (−2.11 to 1.54)
Disabled	6	2 (33.3)	4 (66.7)	0.38 (−1.63 to 2.47)
Declined to state	2	0 (0)	2 (100)	−0.14 (−2.54 to 2.15)
Level of education				
<8th grade	2	0 (0)	2 (100)	−2.36 (−4.34 to −0.31)
9–11th grade	16	3 (18.8)	13 (81.3)	−1.24 (−2.67 to 0.26)
High school or GED	53	14 (26.4)	39 (73.6)	−0.54 (−1.84 to 0.77)
Vocational or technical school	7	1 (14.3)	6 (85.7)	0.03 (−1.19 to 1.28)
Associate degree or some college	38	18 (47.4)	20 (52.6)	0.62 (−0.58 to 1.92)
Bachelor's degree	22	13 (59.1)	9 (40.9)	1.38 (−0.05 to 2.86)
Insurance type				
Medicaid or Medicare	75	19 (25.3)	56 (74.7)	−0.36 (−2.00 to 1.39)
Private insurance	82	40 (48.8)	42 (51.2)	0.05 (−1.63 to 1.78)
Substance use				
No drug use	111	48 (43.2)	63 (56.8)	0.42 (−1.29 to 2.07)
Current drug use	11	1 (9.1)	10 (90.9)	−0.80 (−2.71 to 0.96)
Former drug use	33	10 (30.3)	23 (69.7)	−0.61 (−2.28 to 1.14)
Unknown drug use	4	3 (75)	1 (25)	0.42 (−1.29 to 2.07)
Marital status				
Single	66	20 (30.3)	46 (69.7)	−0.22 (−2.09 to 1.64)
Married	74	32 (43.2)	42 (56.8)	−0.50 (−2.34 to 1.35)
Living as married	11	6 (54.5)	5 (45.5)	0.52 (−1.43 to 2.50)
Divorced	2	2 (100)	0 (0)	0.53 (−1.55 to 2.70)
Separated	4	0 (0)	4 (100)	−0.48 (−2.66 to 1.71)
Method to learn more				
Provider conversation	86	28 (32.6)	58 (67.4)	−0.79 (−1.55 to −0.08)
Pamphlet	40	10 (25)	30 (75)	−1.24 (−2.08 to −0.47)
Prerecorded videos	11	6 (54.5)	5 (45.4)	0.42 (−0.73 to 1.59)
Social media	24	12 (50)	12 (50)	0.55 (−0.38 to 1.56)
Webinar	9	6 (66.7)	3 (33.3)	0.44 (−0.79 to 1.59)
Written on website	38	17 (44.7)	21 (55.3)	0.12 (−0.69 to 0.98)
Infectious disease status				
Received flu vaccine	30	15 (50)	15 (50)	0.18 (−0.59 to 0.95)
Received Tdap vaccine	61	21 (34.4)	40 (65.6)	0.61 (−0.25 to 1.45)
Previous positive COVID test	6	2 (33.3)	4 (66.7)	0.08 (−0.47 to 0.65)

CI, credible interval; COVID-19, coronavirus disease 2019; Tdap, tetanus diphtheria pertussis.

**FIG. 3.**
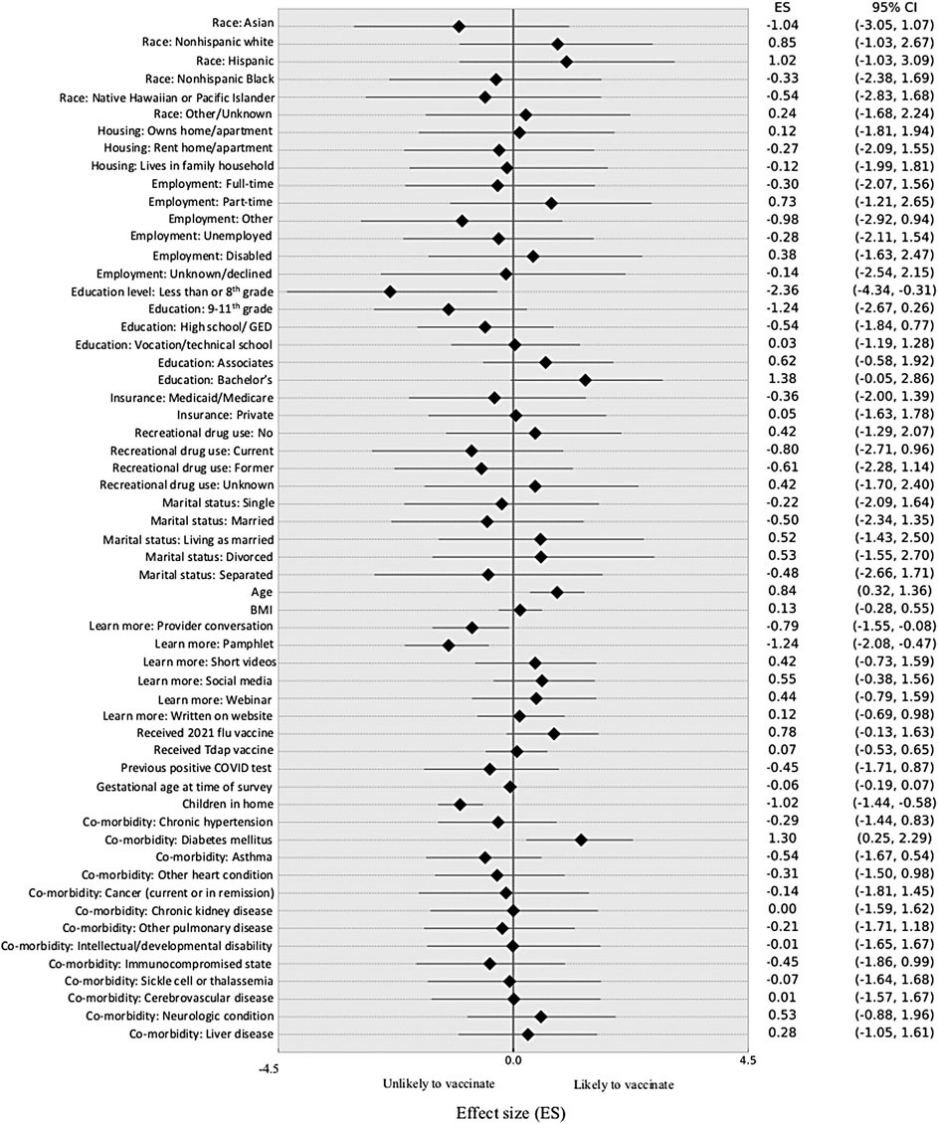
Hierarchical logistic Bayesian regression.

^a^ Bayesian model included in Figure 3.

A hierarchical Bayesian logistic regression was performed to control for multiple variables simultaneously based on conditional probabilities (Fig. 3). This model suggests that vaccinated individuals were slightly older (effect size 0.84 [95% credible interval; CI: 0.32–1.36]) (Fig. 3 and Table 1).

There were no significant differences in race/ethnicity (Asian: −1.04 [95% CI: −3.05 to 1.07], non-Hispanic White: 0.85 [95% CI: −1.03 to 2.67], Hispanic: 1.02 [95% CI: −1.03 to 3.09], non-Hispanic Black: −0.33 [95% CI: −2.38 to 1.69], American Indian/Alaskan Native: −0.54 [95% CI: −2.83 to 1.68], other: 0.24 [95% CI: −1.68 to 2.24]); living situation (own home: 0.12 [95% CI: −1.81 to 1.94], rent home: −0.27 [95% CI: −2.09 to 1.55], live in family household: −0.12 [95% CI: −1.99 to 1.81]); marital status (single: −0.22 [95% CI: −2.09 to 1.64], married: −0.50 [95% CI: −2.34 to 1.35], living as married: 0.52 [95% CI: −1.43 to 2.50], divorced: 0.52 [95% CI: −1.43 to 2.50], separated: −0.48 [95% CI: −2.66 to 1.71]).

There was no difference in employment status (full time: −0.30 [95% CI: −2.07 to 1.56], part time: 0.73 [95% CI: −1.21 to 2.65], other: −0.98 [95% CI: −2.92 to 0.94], unemployed: −0.28 [95% CI: −2.11 to 1.54], disabled: 0.38 [95% CI: −1.63 to 2.47]); insurance type (Medicaid or Medicare: −0.36 [95% CI: −2.00 to 1.39], private insurance: 0.05 [95% CI: −1.63 to 1.78]); history of recreational drug use (no drug use: 0.42 [95% CI: −1.29 to 2.07], current drug use: −0.80 [95% CI: −2.71 to 0.96], former drug use: −0.61 [95% CI: −2.28 to 1.14], unknown drug use: 0.42 [95% CI: −1.29 to 2.07]) (Fig. 3 and Table 1).

There were no differences in pregravid body mass index (0.13 [95% CI: −0.28 to 0.55]), number of living children (−1.02 [95% CI: −1.44 to −0.58]), or gestational age at the time of survey (−0.06 [95% CI: −0.19 to 0.07]) between vaccinated and unvaccinated groups (Fig. 3 and Table 2).

**Table 2.** Continuous Abstracted Information, by Coronavirus Disease 2019 Vaccinated Versus Not Vaccinated Status

Characteristics	Vaccinated: mean (standard deviation)	Not vaccinated: mean (standard deviation)	Bayesian model: effect size (95% CI)^a^
Age, years	30.6 (± 5.6)	28.9 (± 5.8)	0.84 (0.32 to 1.36)
Pregravid body mass index, kg/m^2^	30.9 (± 9.2)	31.4 (± 9.0)	0.13 (−0.28 to 0.55)
Gestational age range, weeks	28 0/7–29 6/7^b^	26 0/7–27 6/7^b^	−0.06 (−0.19 to 0.07)
No. of living children	1 (± 1)	2 (± 2)	−1.02 (−1.44 to −0.58)

^a^ Bayesian model included in Figure 3.

^b^ Standard deviation unable to be calculated.

We identified a trend that patients with less education were less likely to be vaccinated (less than eighth grade completed: −2.36 [95% CI: −4.34 to −0.31], ninth to eleventh grade: −1.24 [95% CI: −2.67 to 0.26], high school or GED: −0.54 [95% CI: −1.84 to 0.77], vocational or technical school: 0.03 [95% CI: −1.19 to 1.28], associate degree or some college: 0.62 [95% CI: −0.58 to 1.92], and bachelor's degree: 1.38 [95% CI: −0.05 to 2.86]) (Fig. 3 and Table 1). Patients with an eighth-grade level education or less were the least likely to have been vaccinated. Vaccination rates increase with increasing level of education.

There was no correlation between COVID-19 vaccine rates and influenza (0.18 [95% CI: −0.59 to 0.95]) or tetanus diphtheria pertussis (Tdap) vaccination rates (0.61 [95% CI: −0.25 to 1.45]) (Fig. 3 and Table 1). Fifty percent of both the COVID-19 vaccinated and unvaccinated groups received the 2021 influenza vaccine. 65.6% of COVID-19 unvaccinated participants received the Tdap vaccine during pregnancy, whereas only 34.4% of COVID-19 vaccinated participants had. Six participants had a previous positive COVID-19 test on file; four of these participants remained unvaccinated at the time of survey (0.08 [95% CI: −0.47 to 0.65]) (Fig. 3 and Table 1). This was not statistically significant, likely because of the small number of participants with a positive test.

The high-risk clinic population was selected to capture pregnant patients with medical comorbidities. Conditions that qualified an individual for early vaccination during the stratified rollout in New York Status were evaluated. It was hypothesized that vaccination rates would be higher in these high-risk individuals. Diabetes was the only medical comorbidity associated with a higher vaccination rate (1.30 [95% CI: 0.25–2.29]) (Fig. 3 and Table 3).

**Table 3.** Medical Comorbidities by Vaccinated Versus Unvaccinated Status, by Coronavirus Disease 2019 Vaccinated Versus Not Vaccinated Status

Characteristics	Total (%)	Vaccinated, *n* (%)	Not vaccinated, *n* (%)	Bayesian model: effect size (95% CI)^a^
Hypertension	20	6 (30.0)	14 (70.0)	−0.29 (−1.44 to 0.83)
Diabetes mellitus	27	16 (59.3)	11 (40.7)	1.30 (0.25 to 2.29)
Asthma	40	12 (30.0)	28 (70.0)	−0.54 (−1.67 to 0.54)
Other heart conditions	9	2 (22.2)	7 (77.8)	−0.31 (−1.50 to 0.98)
Cancer	1	0 (0)	1 (100)	−0.14 (−1.81 to 1.45)
Chronic kidney disease	0	0	0	0.00 (−1.59 to 1.62)
Other pulmonary disease	3	1 (33.3)	2 (66.7)	−0.21 (−1.71 to 1.18)
Intellectual and developmental disabilities	0	0	0	−0.01 (−1.65 to 1.67)
Immunocompromised states	6	2 (33.3)	4 (66.7)	−0.45 (−1.86 to 0.99)
Sickle cell disease or thalassemia	1	0 (0)	1 (100)	−0.07 (−1.64 to 1.68)
Cerebrovascular disease	0	0	0	0.01 (−1.57 to 1.67)
Neurological conditions	4	2 (50.0)	2 (50.0)	0.53 (−0.88 to 1.96)
Liver disease	7	3 (42.9)	4 (57.1)	0.28 (−1.05 to 1.61)

^a^ Bayesian model included in Figure 3.

No other medical comorbidity correlated with COVID-19 vaccination status (hypertension: −0.29 [95% CI: −1.44 to 0.83], asthma: −0.54 [95% CI: −1.67 to 0.54], other heart conditions: −0.31 [95% CI: −1.50 to 0.98], cancer: −0.14 [95% CI: −1.81 to 1.45], chronic kidney disease: 0.00 [95% CI: −1.59 to 1.62], other pulmonary disease: −0.21 [95% CI: −1.71 to 1.18], intellectual and developmental disabilities: −0.01 [95% CI: −1.65 to 1.67], immunocompromised states: −0.45 [95% CI: −1.86 to 0.99], sickle cell disease or thalassemia: −0.07 [95% CI: −1.64 to 1.68], cerebrovascular disease: 0.01 [95% CI: −1.57 to 1.67], neurological conditions: 0.53 [95% CI: −0.88 to 1.96], and liver disease: 0.28 [95% CI: −1.05 to 1.61]) (Fig. 3 and Table 3).

**FIG. 4.**
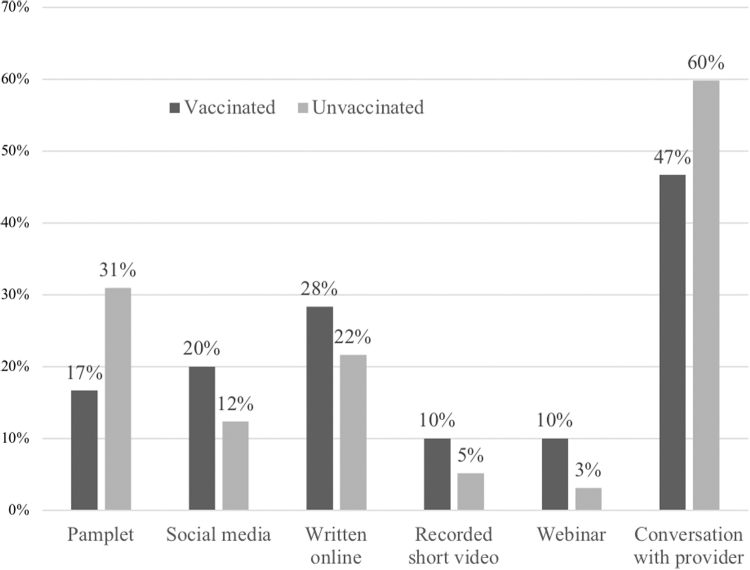
Patients answered how they would prefer to learn more about vaccinations.

When given different options to learn more about the vaccine, most patients in both groups preferred to learn more about the vaccine *via* a conversation with their doctor (Fig. 4).

## Discussion

The vaccination rate of our cohort is higher than the published national vaccination rates in pregnant women^3^ although is still low. A lack of data in pregnancy is the most common cited reason for being unvaccinated. Despite strong recommendations by the experts in the field and subsequent studies demonstrating safety in pregnancy,^14,28–30^ patients continue to express this concern. The exclusion of pregnant women in the first COVID-19 vaccination trials^1,12^ propagated a sentiment of fear and danger that persists. An element of politics also appeared to be influencing the decision to get vaccinated or not. However, this study was not designed to address this issue and currently cannot be quantified.

Less formal education correlates with a decrease in vaccination. This is consistent with international trends of COVID-19 vaccination hesitancy,^36,37^ but unlike vaccination trends seen with childhood vaccination.^38^ Increasing age is associated with increased COVID-19 vaccination rates. This may represent patients who have completed more formal education. It is unlikely that patients with a less formal education prioritize reading the latest scientific information about COVID-19 vaccination in pregnancy; this may impact the trend seen.

Influenza vaccination is recommended universally to pregnant patients due to concern for increased severe illness from influenza.^39^ Tdap vaccination is recommended to pregnant patients starting at 28 weeks of gestation to aid with immunity against pertussis in mother and neonate after delivery.^39^ Influenza and Tdap vaccination statuses are unrelated to COVID-19 vaccination status. This suggests that the fears and concerns surrounding COVID-19 vaccinations are not universal.

Patients with a history of diabetes mellitus are associated with an increase in vaccination. Unlike other comorbidities, patients with diabetes mellitus require more frequent visits with a provider throughout pregnancy independent of prepregnancy control of disease to adjust medications and perform adequate fetal monitoring.^40^ Perhaps the frequent visits with a provider can positively contribute to a patient's understanding and willingness to be vaccinated.

Most patients in both groups prefer to learn about the vaccination *via* a conversation with their provider. This outlines the importance of the providers' responsibility to include well-informed counseling about the COVID-19 vaccine in routine visits.

This study is limited by the small sample size. The timeframe of survey collection was limited due to research staff availability and as a result, the audience captured is small. Nevertheless, we believe that it reflects the true rates of COVID-19 vaccination in central New York. The nature of the survey was nonrandom as every patient who presented to the clinic while a research staff member was present was included. The survey was available only in English; the generalizability of these results is limited to those with English proficiency.

The survey was available only *via* tablet and may underrepresent participants who are uncomfortable with technology, although the goal of the staff member at time of survey administration was to attempt to reduce this. Although the staff member was blinded to the answers, the presence of the staff member may increasingly contribute to a social desirability bias. Recall bias is present as the survey was not administered at the time of potential COVID-19 vaccination. The high-risk maternal fetal medicine clinic was targeted to capture a higher-risk pregnant population, but the result is a Berkson bias.

Anecdotally, several patients included in this survey have been subsequently admitted while pregnant due to symptoms secondary to COVID-19 pneumonia. It would be of interest to follow up with patients to expand on number of provider visits in the past year, and if there is any information that is influencing their opinions about the vaccine since the initial survey. It would also be meaningful to evaluate if an intervention, such as provider education and talking points for provider-led conversations at routine visits, leads to a change in vaccine rates. COVID-19 vaccination opinions are dynamic throughout the natural history of the pandemic and future studies may be useful to re-evaluate patients' evolving views.

## Abbreviations Used

CDC: Centers for Disease Control
CI: credible interval
COVID-19: coronavirus disease 2019
Tdap: tetanus diphtheria pertussis
